# The Influence of Playing Position on Physical, Physiological, and Technical Demands in Adult Male Soccer Matches: A Systematic Scoping Review with Evidence Gap Map

**DOI:** 10.1007/s40279-024-02088-z

**Published:** 2024-09-11

**Authors:** Hugo Sarmento, Diogo V. Martinho, Élvio R. Gouveia, José Afonso, Paweł Chmura, Adam Field, Nestor Ordoñez Savedra, Rafael Oliveira, Gibson Praça, Rui Silva, Joel Barrera-Díaz, Filipe Manuel Clemente

**Affiliations:** 1 University of Coimbra, Research Unit for Sport and Physical Activity, Faculty of Sport Sciences and Physical Education, Coimbra, Portugal; 2 LARSYS, Interactive Technologies Institute, Funchal, Portugal; 3 Department of Physical Education and Sport, University of Madeira, Funchal, Portugal; 4 CIPER, Faculdade de Motricidade Humana, Universidade de Lisboa, Lisbon, Portugal; 5 Faculty of Sport, Centre of Research, Education, Innovation, and Intervention in Sport (CIFI2D), University of Porto, Porto, Portugal; 6 Department of Team Games, Wroclaw University of Health and Sport Sciences, 51-612 Wrocław, Poland; 7 Department of Sport and Exercise Science, Institute of Sport, Manchester Metropolitan University, Manchester, UK; 8 Research Group in Sports Science and Physical Activity, Faculty of Health Sciences, Sports Science Program, University of Applied and Environmental Sciences, Bogota, Colombia; 9 Santarém Polytechnic University, School of Sport, Rio Maior, Portugal; 10 Research Center in Sport Sciences, Health Sciences and Human Development (CIDESD), Santarém Polytechnic University, Rio Maior, Portugal; 11 Sports Department/UFMG Soccer Science Center/CECA, Universidade Federal de Minas Gerais, Belo Horizonte, Brazil; 12 Escola Superior Desporto e Lazer, Instituto Politécnico de Viana do Castelo, Rua Escola Industrial e Comercial de Nun’Álvares, Viana do Castelo, Portugal; 13 Sport Physical Activity and Health Research & Innovation Center, 4900-347 Viana do Castelo, Portugal; 14 Gdansk University of Physical Education and Sport, 80-336 Gdańsk, Poland

## Abstract

**Background:**

There has been an increase in studies examining the demands of soccer relative to each playing position in recent years. Understanding the physical, physiological, and technical demands on soccer players according to their positional role during competitive matches is necessary to understand match requirements and develop position-specific training practices. Thereby, there is a clear need to synthesize the information on the different profiles of each playing position.

**Objective:**

This review aimed to organize the literature investigating physical, physiological, and technical demands according to playing positions and provide a framework to identify gaps and suggestions for future studies.

**Methods:**

A systematic search was conducted in October 2023 using four electronic databases: Web of Science, SPORTDiscus, PubMed and Scopus. The review followed PRISMA 2020 guidelines and the PRISMA-ScR extension for Scoping Reviews. The studies were included if the sample comprised adult male soccer players categorized from Tier 3 to Tier 5 (i.e., highly trained/national level, elite/international level, or world class) and compared the physical, physiological, or technical parameters across playing positions.

**Results:**

A total of 178 studies met the inclusion criteria and were included in the review. The number of teams, players, and matches analyzed per study varied considerably. Although a range of classifications were reported across studies, 59% of studies classified players as central defenders, full-backs, central midfielders, wide midfielders, and forwards. The findings suggests that central and external midfielders, and external defenders cover greater total and high-speed distance than forwards or central defenders. Sprint distance was higher in external midfielders versus all other positions. Defenders and central midfielders perform more passes than external midfielders and forwards. Heart rate was the most commonly reported physiological variable across playing positions. When expressed as a percentage of maximal heart rate, midfielders presented higher mean values than all other playing positions.

**Conclusion:**

This scoping review demonstrates that there are differences in the demands on players across playing positions in soccer. Training practices in soccer should be based on the specific requirements of each positional role to ensure players can fulfill their tactical responsibilities during the game.

**Supplementary Information:**

The online version contains supplementary material available at 10.1007/s40279-024-02088-z.

## Key Points

**Table:** 

Central midfielders, and external midfielders and defenders cover greater total and high-speed distance than forwards or central defenders.
External midfielders cover greater sprint distances versus all other positions.
Defenders and central midfielders perform more passes than external midfielders and forwards.
Practitioners might individualize training practices where appropriate for each playing position to optimize performance.

## Introduction

Following modern developments in technology, studies of match activity profiles and physiological characteristics have increased over the last 2 decades [1–7]. However, although a high proportion of match-analysis data are provided for an entire team or group of players, there are some studies which have distinguished between different playing positions. To the best of the authors’ knowledge, though, there is currently no high-quality review article synthesizing match performance outputs with a specific emphasis on playing positions. Therefore, despite the rapidly evolving knowledge of soccer and the influx of research articles in the area, there is an existing uncertainty as to whether and how the demands differ between positional roles [8–11]. Given that a high-quality central resource summarizing information in this area fails to exist, the impact of playing positions on the demands of soccer might be overlooked by practitioners. This might result in the prescription of generalized training practices that lack specificity rather than implementing protocols for individual positions.

There is currently a lack of consensus surrounding the grouping of positions and the differences in terminology adopted across studies. For instance, there are studies that group players into three categories: defenders, midfielders, and forwards [12]. Some studies have used four position classification systems that grouped players into defenders, central midfielders, wing midfielders, and forwards [13]. Separate classifications have included six-player systems, dividing players into wide defenders, central defenders, central midfielders, attacking midfielders, wing midfielders, and forwards [14]. However, the most used classification system includes a breakdown of five positions into wide defenders, central defenders, wide midfielders, central midfielders, and forwards/attackers [15–17]. This might be problematic for data accuracy as the distinct classification systems adopted in each study might affect the interpretation of the results [18–21]. Critics have also argued that systems of classification may not be effective in capturing the differences between positions as tactical approaches are manipulated by modern day coaches, and as such, there are a greater number of playing positions than previous studies suggest. For instance, a recent investigation reported a larger quantity of positions, depending on whether teams play a more attacking or defensive system [22]. With the contrasting interpretations and methodological approaches, clarity on precisely which conclusions can be drawn would support scientific development and assurances around applied practice.

Considering the need to enhance understanding of position-specific roles, it appears necessary to systematically review and synthesize the available information concerning physical, physiological, and technical assessments in soccer players, according to specific playing positions. Therefore, the present scoping review aimed to map (1) the impacts of playing position on physical, physiological, and technical demands in adult male soccer matches; (2) understand existing analyses that explain players’ activity profiles in different contexts; and (3) identify literature gaps and make suggestions for further research.

## Methods

This scoping review was developed according to the PRISMA 2020 guidelines [23] and the PRISMA-ScR extension for Scoping Reviews [24].

### Protocol and Registration

The scoping review was registered on the Open Science Framework (https://osf.io/6gkaw/) on 24 October, 2023.

### Eligibility Criteria

This systematic review included all original studies that had been published or were available ahead of print, without imposing any language or date restrictions. The inclusion criteria were guided by the Participants, Intervention/Exposure, Comparators, Outcomes, and Study Design (PICOS/PECOS) framework as follows: (1) Participants—healthy adult male soccer players categorized from Tier 3 to 5 based on the Participation Classification Framework [25]; (2) Intervention/Exposure—any intervention or exposure relevant to the demands experienced during matches; (3) Comparators – optional; (4) Outcomes—physical measures (such as locomotor demands at different intensity thresholds and mechanical demands at different intensities), physiological measures (such as heart rate responses, blood lactate levels, and oxygen uptake), and technical assessments (such as the frequency of defensive or attacking actions); (5) Study Design—no restrictions on the types of study designs eligible for inclusion.

### Information Sources and Search Strategy

The following databases were searched: PubMed, Scopus, SPORTDiscus and Web of Science (all databases). These searches encompassed relevant publications available up to 25 October 2023. In addition, manual searches were conducted on the reference lists of the included studies to identify potentially relevant studies. Snowballing citation tracking in Web of Science was also conducted, in addition to consultation with two externals with knowledge in the current area to determine whether any studies had not been identified.

The search strategy was as follows:

[Title]: football* OR soccer

AND

[Title/abstract] match* OR game* OR competit* OR “match-play”

AND

[Full text]: “time-motion” OR demand* OR running* OR locomotor OR mechanic* OR technic* OR tactic* OR performance* OR physical OR Physiologic* or Neuromuscular

AND

[Full text] formation* OR position*

The search strategy is detailed in Table 1.

**Table 1:** Full search strategy for each database

PubMed	(((football*[Title] OR soccer[Title]) AND ("match*"[Title/Abstract] OR "game*"[Title/Abstract] OR "competit*"[Title/Abstract] OR "match-play"[Title/Abstract])) AND ("time-motion" OR "demand*" OR "running*" OR locomotor OR "mechanic*" OR "technic*" OR "tactic*" OR "performance*" OR physical OR Physiologic* or Neuromuscular)) AND ("formation*" OR "position*")
Scopus	(TITLE (football* OR soccer) AND TITLE-ABS-KEY (match* OR game* OR competit* OR "match-play") AND ALL ("time-motion" OR demand* OR running* OR locomotor OR mechanic* OR technic* OR tactic* OR performance* OR physical OR physiologic* OR neuromuscular) AND ALL (formation* OR position*))
SPORTDiscus	TI (football* OR soccer) AND AB (match* OR game* OR competit* OR “match-play”) AND TX ( “time-motion” OR demand* OR running* OR locomotor OR mechanic* OR technic* OR tactic* OR performance* OR physical OR Physiologic* or Neuromuscular) AND TX ( formation* OR position*)
Web of Science	football* OR soccer (Title) AND “match*” OR “game*” OR “competit*” OR “match-play” (Abstract) AND “time-motion” OR “demand*” OR “running*” OR locomotor OR “mechanic*” OR “technic*” OR “tactic*” OR “performance*” OR physical OR Physiologic* or Neuromuscular (All Fields) AND “formation*” OR “position*” (All Fields)

### Selection Process

An automated procedure was executed utilizing EndNote™ 20.6 for Mac (Clarivate™) to remove duplicate records. Manual screenings were also carried out to ensure duplicates were omitted for precision. Two independent reviewers (HS and DM) then examined the titles, abstracts, and reference lists of each study to identify the relevant studies. Full studies were subsequently screened to determine their relevance to the selection criteria. Where discrepancies occurred, a third external reviewer (FMC) was consulted to ensure agreement by consensus.

### Data Extraction

Two investigators (HS and JB) conducted the data extraction process, systematically collecting information according to a predefined template. A third investigator (FC) cross-verified the extracted data to ensure accuracy and reliability. This structured approach to data extraction was facilitated through a comprehensive datasheet that encompassed all pertinent details and essential information.

### Data Items

For each study, a comprehensive set of data was gathered, encompassing various aspects related to the participants, their competitive level, match details, competition type, time of year the study was undertaken, competition location, and country. Regarding playing formation, the collected information encompassed playing positions, their classification, and the methodology employed for classification. For the physical data, a wide range of parameters was considered, including but not limited to distances covered at various speed thresholds, accelerations, and decelerations. These measures were recorded irrespective of whether they were presented as absolute values, per-match figures, or standardized relative to time or designated periods. The tools and systems used for data collection, including specific measurement instruments, were also integrated into the analysis.

Physiological outcomes (e.g. maximal oxygen uptake, heart rate, ratings of perceived exertion) were retrieved when reported as mean ± standard deviation. Technical analysis involved the comprehensive inclusion of both offensive and defensive events, irrespective of their presentation in absolute terms or standardized formats. Detailed information about the data collection instruments employed was also incorporated, along with the validity assessments if manual observations were employed. The characterization of competitive level followed the Participant Classification Framework [25].

### Study Risk of Bias Assessment

The risk of bias was independently assessed by two authors (RO and RS). In the case of disagreements, a third author (HS) made the final decision. The Quality Assessment Tool for Observational Cohort and Cross-Sectional Studies [26] was used to assess the risk of bias in each individual study (Electronic Supplementary Material [ESM] 1). The tool includes questions related to the research question, study population, groups recruited from the same population and uniform eligibility criteria, sample size justification, exposure assessed prior to outcome of measurement, sufficient timeframe to observe an effect, different levels of the exposure effect, exposures measurement, repeated exposure assessment, outcome measurement(s), blinding of outcomes assessors, follow-up rate, and statistical analyses.

## Results

### Study Identification and Selection

**Fig. 1:**
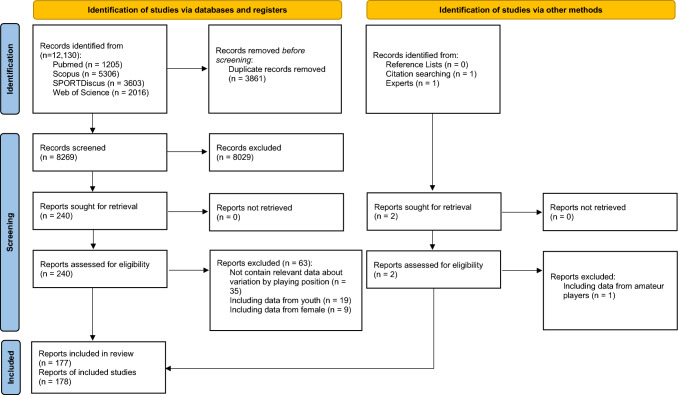
Flow chart of the study selection procedures used in the present review article (*n* = 178)

The initial searches returned 12,130 studies. Duplicates were then removed (3861 studies) automatically using Reference Manager software (EndNote™, version 20.6, Clarivate Analytics, Philadelphia, PA, USA). The titles and abstracts of each study (8269 studies) were then screened for their relevance, which resulted in the rejection of 8029 studies. The full texts of the remaining 240 studies were analyzed considering the eligibility criteria, and 63 papers were removed for the following reasons: data about playing position variation were not present (*n* = 35), samples used youth (*n* = 19) or combined male and female adult soccer players (*n* = 9). One further eligible reference was identified by other methods. In total, 178 papers met the criteria and were included in the final scoping review [2, 6, 8–15, 18–22, 27–189] (see Fig. 1).

### Study Characteristics

**Fig. 2:**
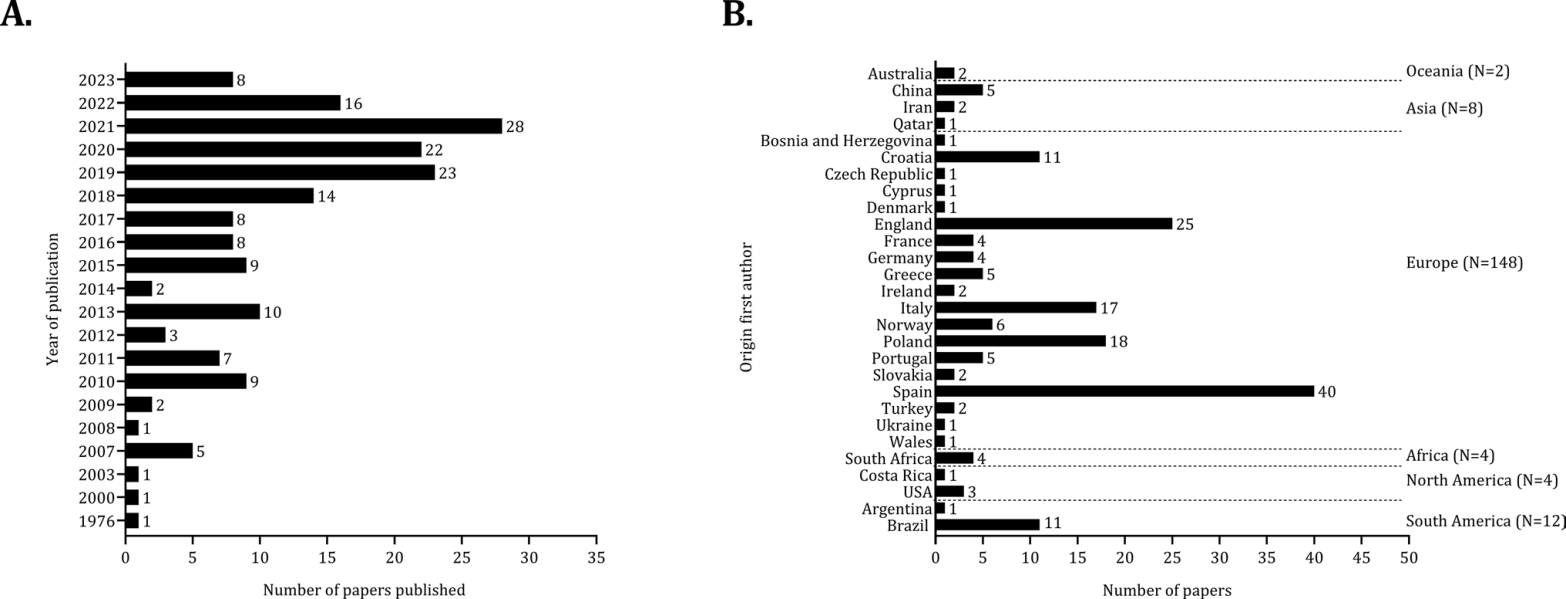
Number of papers published by year (**A**) and according to the origin of the first author (**B**)

**Fig. 3:**
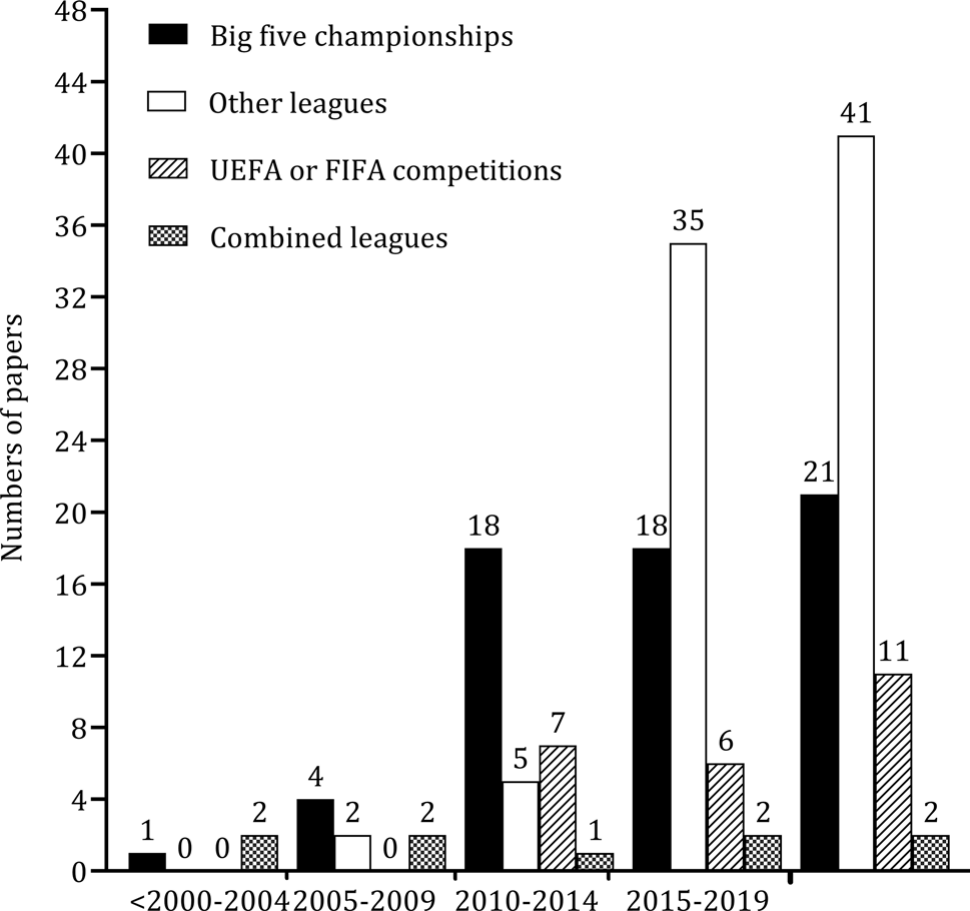
Number of studies grouped by competitive league. *FIFA* Federation Internationale de Football Association, *UEFA* Union of European Football Associations

Figure 2 presents the frequencies of the studies by year of publication (A). Of the 178 papers, 54% (*n* = 96) were published in the last 5 years (2019–2023). The examination of physical, physiological, and technical characteristics according to playing position was prevalent after 2018, whilst a negligible number of papers were published between 1976 and 2007. In the years 2001, 2002, 2004, 2005, and 2006, no studies were published on this topic. Figure 2 (B) shows the distribution of studies per country of origin of the first author. The affiliation of the first author was in Europe in more than 80% of papers (*n* = 148). The most frequent countries that the first authors were affiliated with were Spain (*n* = 41; 27%) and England (*n* = 25; 15%). The percentages of first authors affiliated with other continents were trivial: South America (*n* = 12; 6%), North America (*n* = 4; 2%), Africa (*n* = 4; 2%), Asia (*n* = 8, 4%), and Oceania (*n* = 2; 1%). The number of studies published according to the type of competition analyzed by 5-year periods presented in Fig. 3. There was an exponential increase in publications after 2010 in all competitions. The number of publications in the most popular soccer competitions (Spain, France, Germany, Italy, England) was stable from 2010, while, in other leagues from 2010 to 2023, the rate of studies considering variation by playing position increased by ~ 75%.

**Fig. 4:**
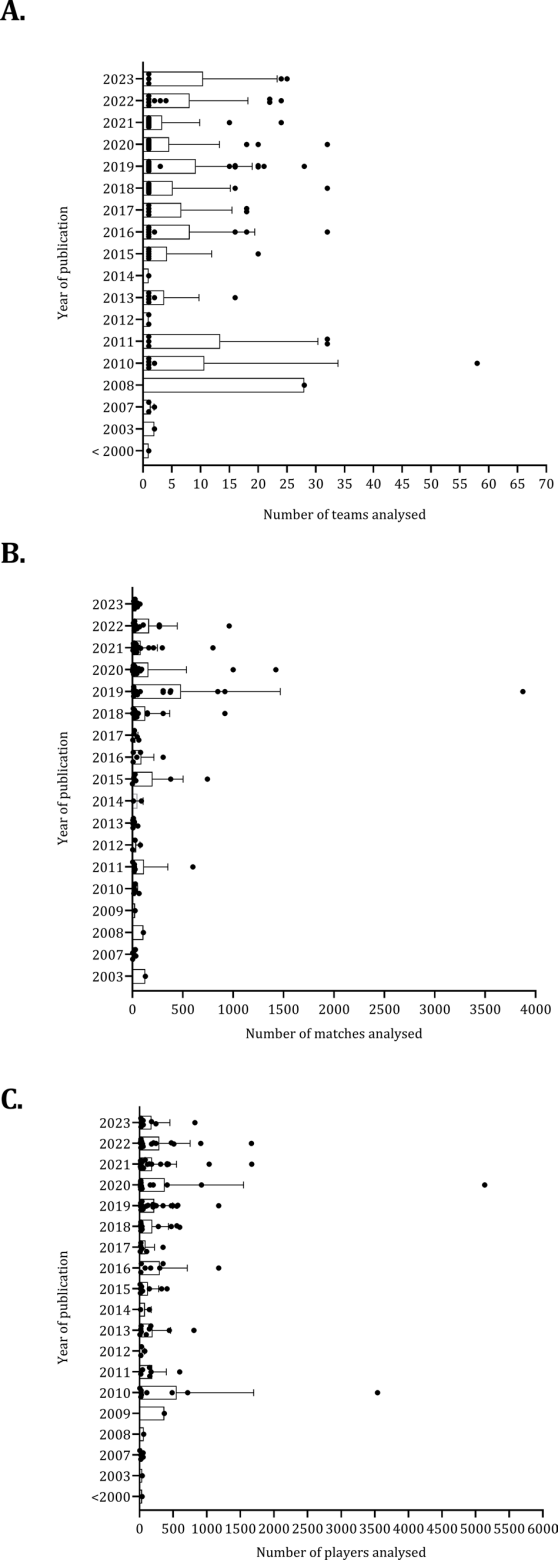
Number of teams (**A**), matches (**B**), and players (**C**) for each study

The characteristics of the studies (number of teams, matches, and players) are summarized in Fig. 4 (A, B, and C). This information was not reported in all studies included in the current review. The number of teams analyzed has increased since 2010, but a substantial variation exists across studies (A). The maximum number of teams analyzed was found in a paper that included data from 20 countries and 58 teams [10]. The number of matches analyzed was comparable between studies (B). A comparison of the Spanish and English elite competitions was conducted in 600 matches played in the 2006–2007 season [67]. The description of the total distance covered, and distance covered at different intensities in the Bundesliga from 2014 until 2016 was examined in 918 matches [15]. A longitudinal study of goalkeepers from the Spanish La Liga observed 3874 matches across six consecutive sessions [161]. The number of players examined was consistent between before 2000–2023 (C). Two studies investigated a substantial number of players, 3540 [68] and 5136 [182].

### Methodological Characteristics

**Fig. 5:**
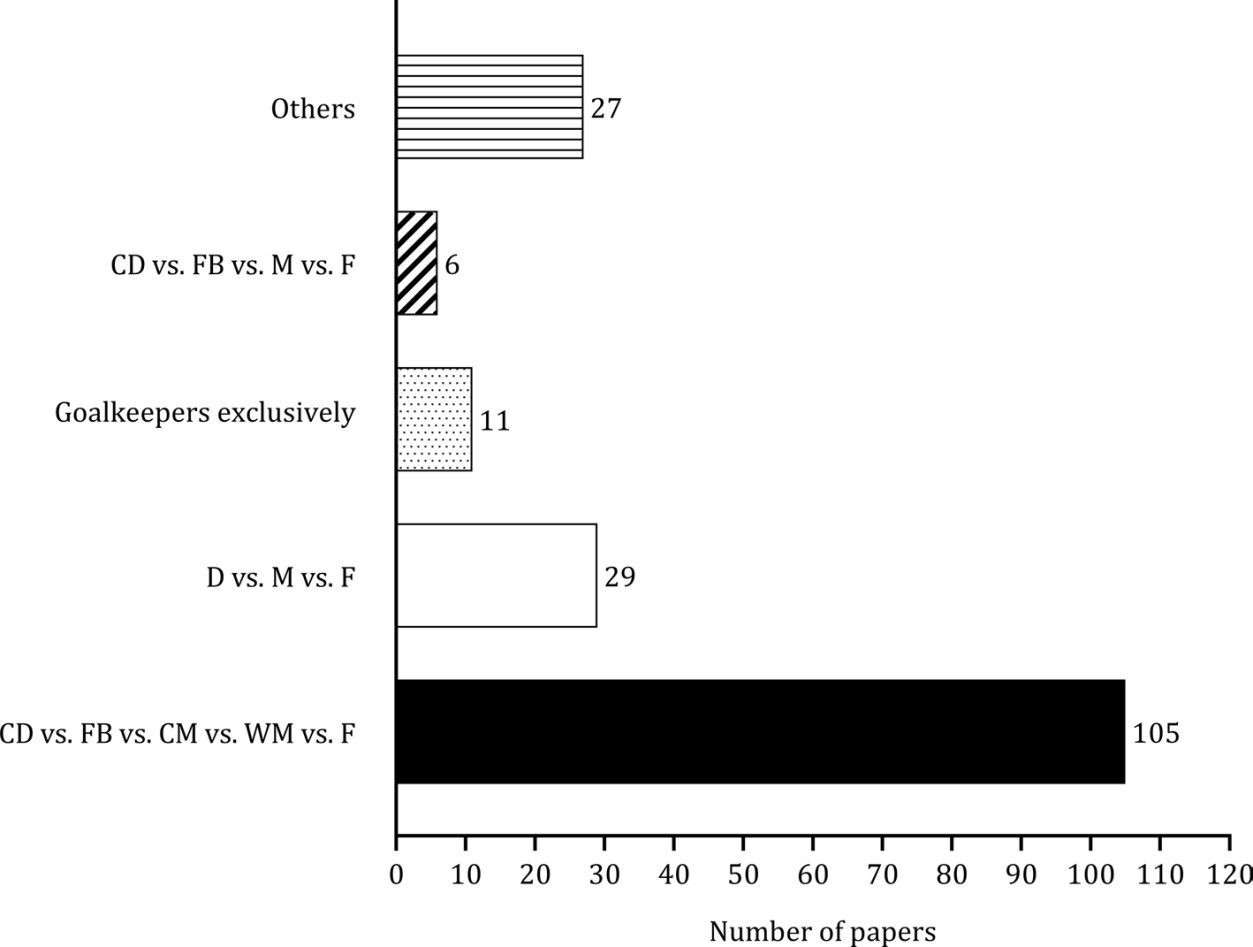
Frequency of papers considering different positional groups. *CD* central defender, *CM* central midfielder, *D* defender, *F* forward, *FB* full-back, *M* midfielder, *WM* wide midfielder

**Fig. 6:**
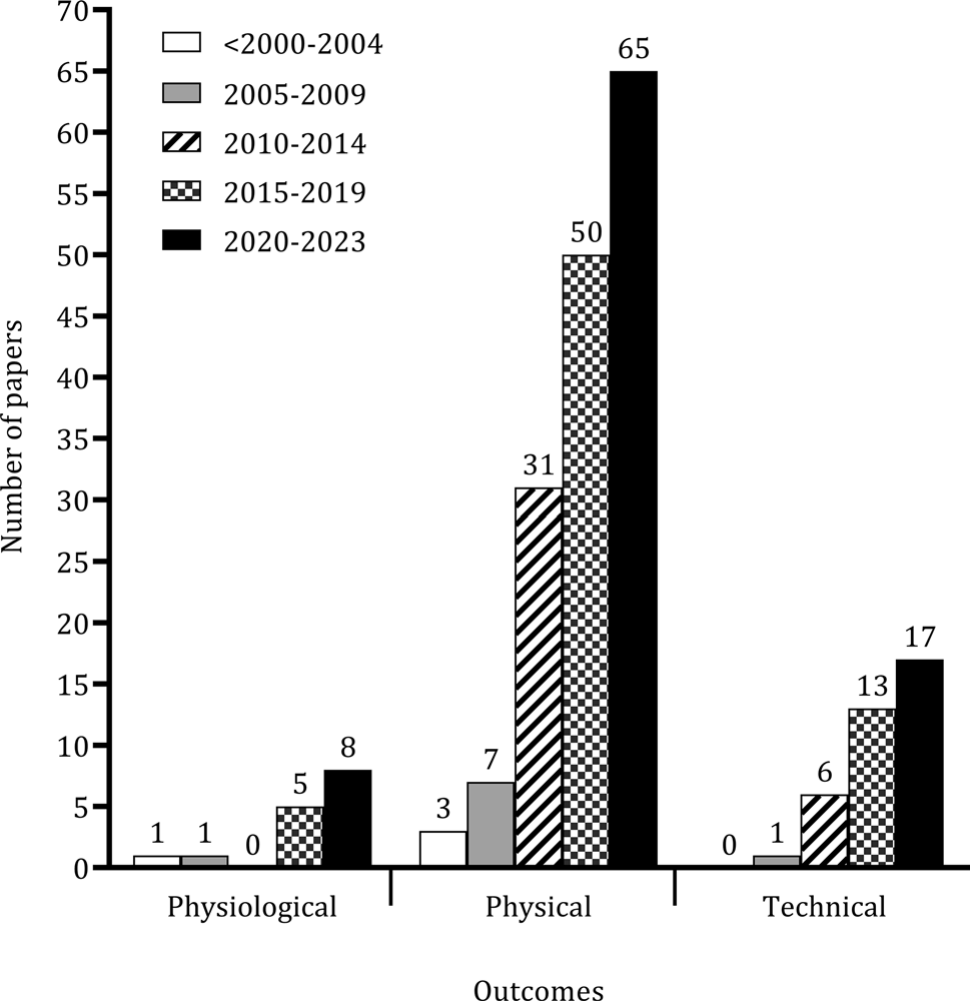
Number of papers (grouped in 5-year periods) about physiological, physical, and tactical/technical parameters

Figure 5 describes the classifications used when players are grouped by playing position. Of the 178 studies, 59% (*n* = 105) classified players as central defenders, full-backs, central midfielders, wide midfielders and forwards, while 16% (*n* = 29) ignored full-back and external midfielder groups. The main topics of the papers were grouped into three categories (physiological, physical, technical), as illustrated in Fig. 6. Some studies aimed to examine variation by playing position in multiple topics. Although there was an increase in the number of studies on different topics from  before 2000, the number of studies investigating physiological parameters was negligible. Physical parameters associated with match performance were frequently examined after 2010. Automatic or semi-automatic video-based systems and microelectromechanical systems were used in 78 and 66 studies, respectively, to measure activity during soccer matches. Total distance covered (*n* = 109), high-speed running (*n* = 62), and sprinting (*n* = 73) were the most reported variables. Few studies (*n* = 9) focused on physiological parameters when players were examined by playing position. Seven studies measured mean heart rate. Video-based systems and notational analyses were the most frequent methods to investigate technical actions (see Table 2).

**Table 2:** Measurement of the main physical, physiological, technical, and tactical variables in adult male soccer matches

Main outcome	Physical	Physiological	Technical	Tactical
Instruments	MEMS (*n* = 66) VBTS (*n* = 78) NS (*n* = 6)	PBI (*n* = 9)	VBTS (*n* = 18) NS (*n* = 12)	VBTS (*n* = 2) NS (*n* = 12)
Measures	Total distance (*n* = 109) High-speed running (*n* = 62) Sprinting (*n* = 73)	Mean heart rate (*n* = 7) RPE (*n* = 2)	^a^	^a^

*MEMS* microelectromechanical systems, *NS* notational system, *PBI* physiological-based instruments, *RPE* rate of perceived exertion, *VBTS* video-based tracking systems

^a^Different variables were analyzed

**Fig. 7:**
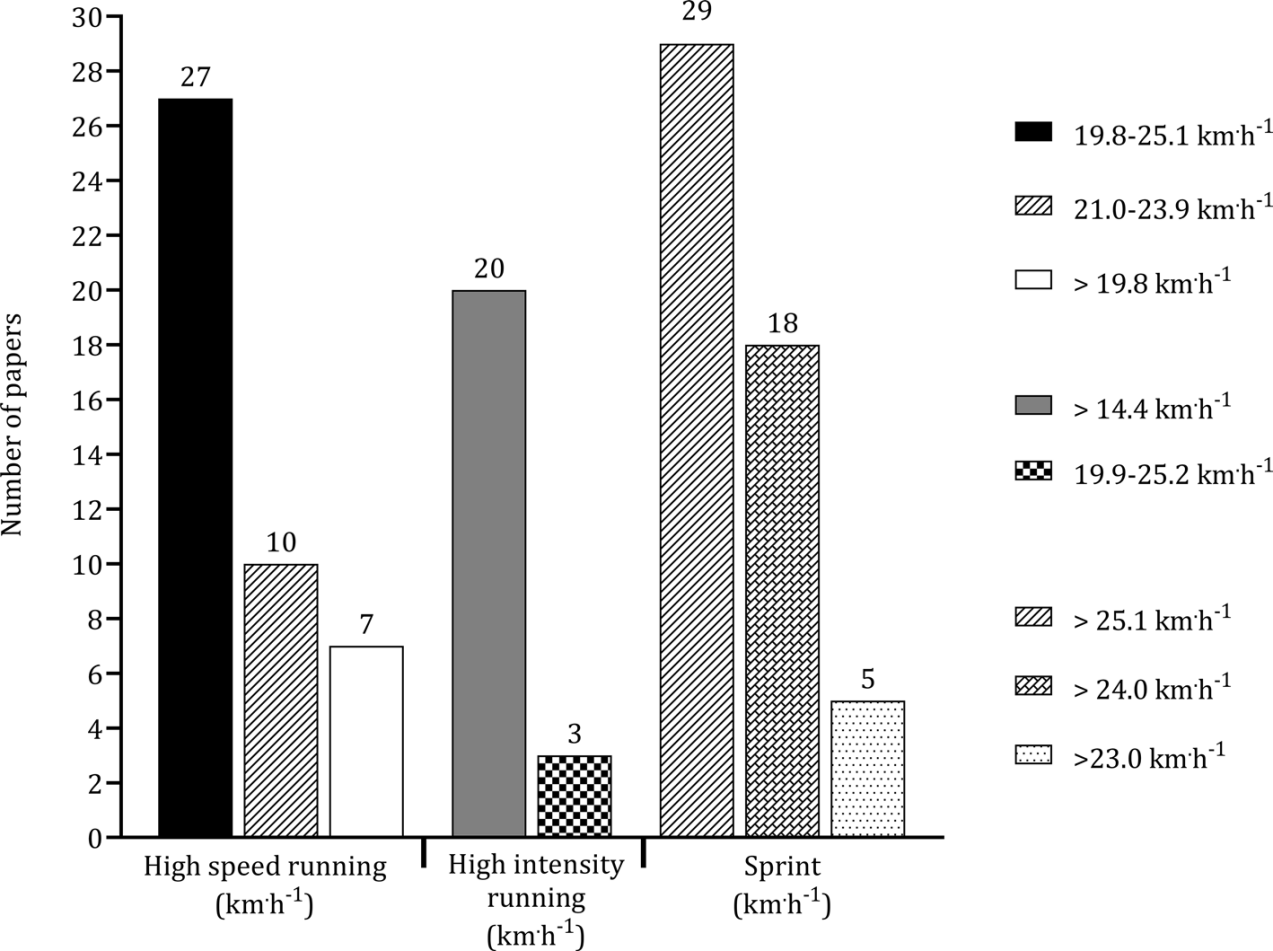
Number of papers that examined high-speed running, high-intensity running, and sprinting. The frequency of papers was organized according to the most reported thresholds

ESM 2 presents the running thresholds collected from the studies in the present review. Multiple running speed thresholds have been used in soccer players. Figure 7 presents the most frequent thresholds in the literature, which include ‘high-speed running’ (19.8 km^.^h^−1^–25.1 km^.^h^−1^), ‘high-intensity running’ (> 14.4 km^.^h^−1^) and ‘sprinting’ (> 25.1 km^.^h^−1^).

### Results of Included Studies

**Fig. 8:**
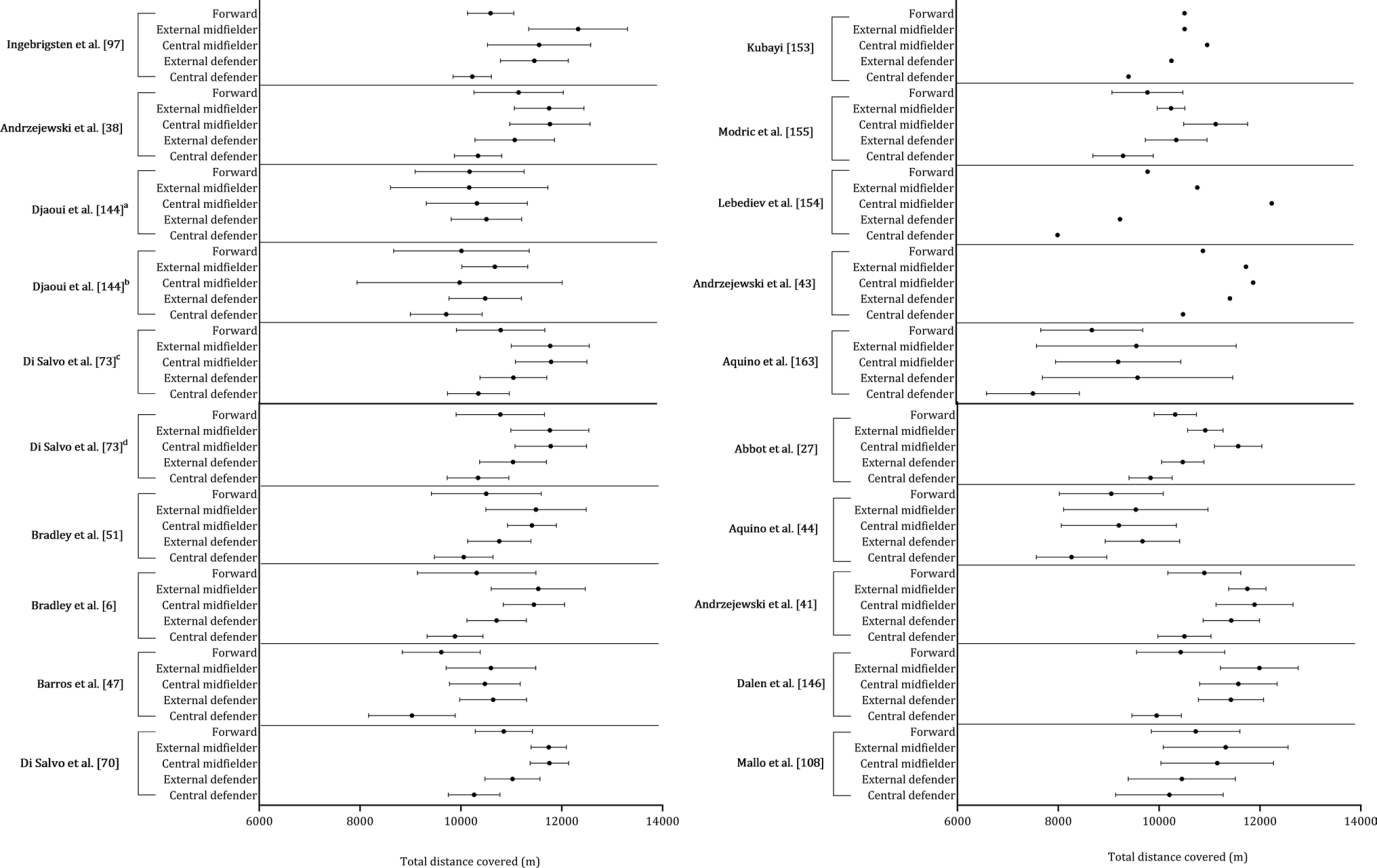

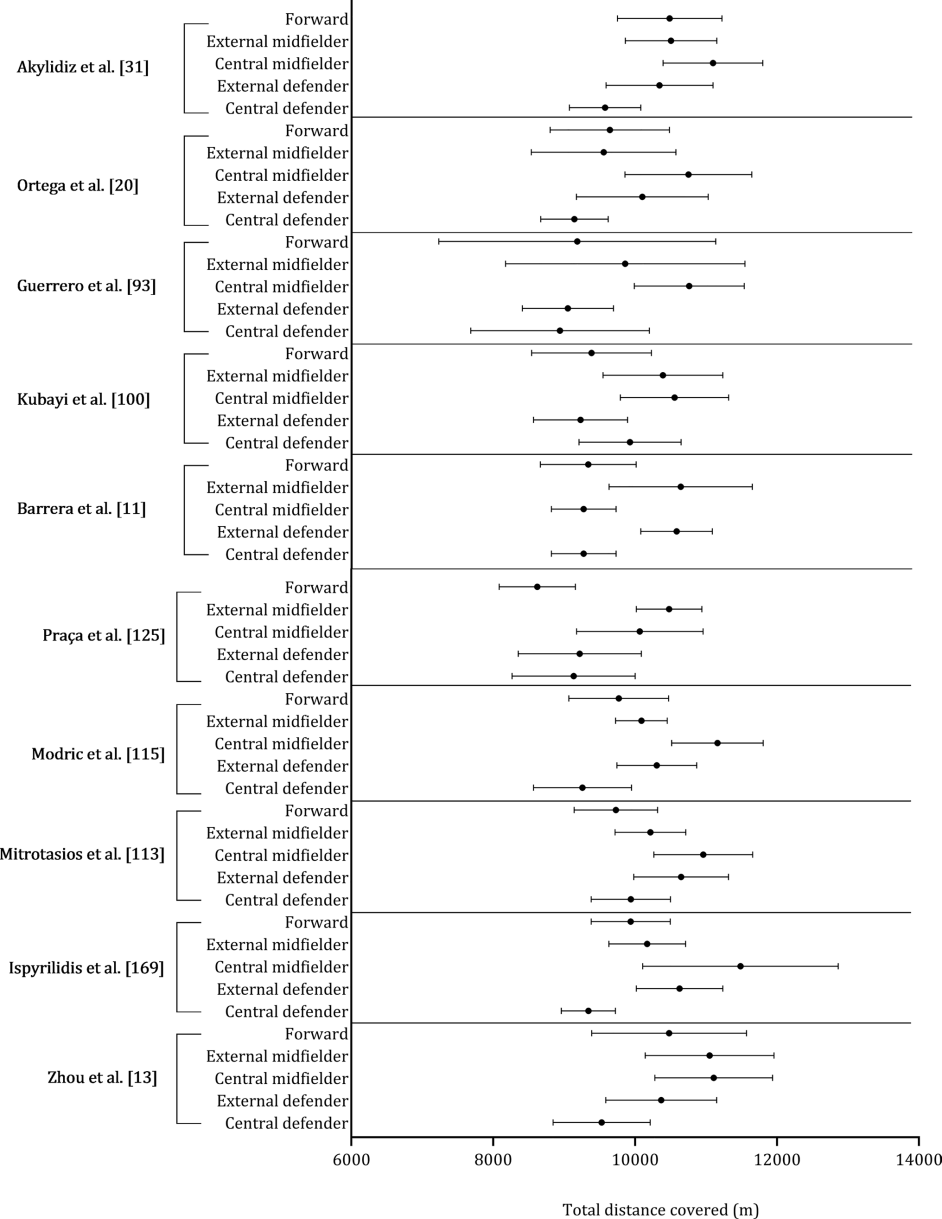
Descriptive statistics (mean ± standard deviation) of total distance covered by playing position (central defender, external defender, central midfielder, external midfielder, forward). ^a^non-congested fixture, ^b^congested fixture, ^c^Championship League, ^d^Premiership

**Fig. 9:**
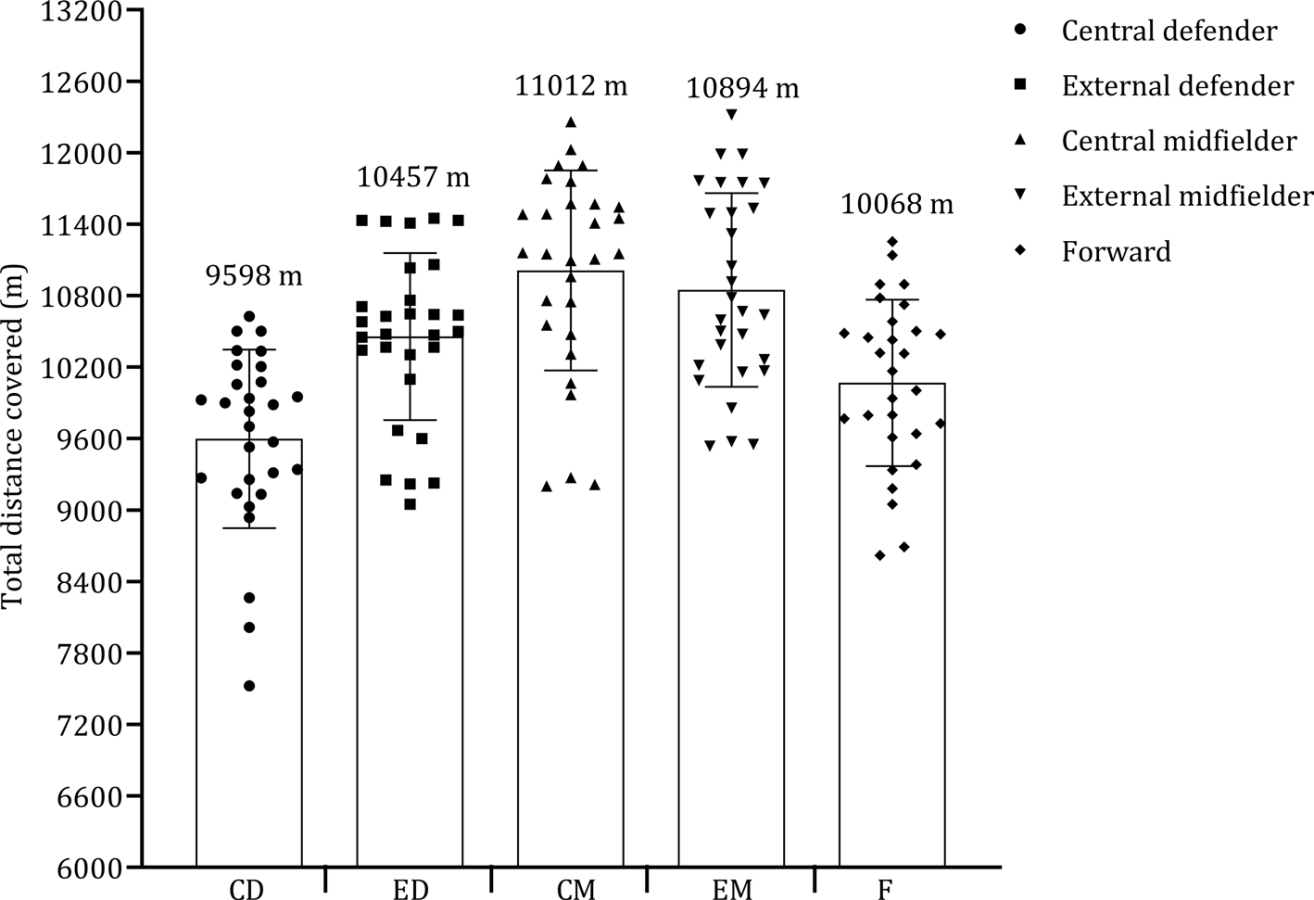
Pooled mean and standard deviation of studies that examined total distance covered by playing position. Each symbol represents the mean of individual studies

Figure 8 shows the mean ± standard deviation distance covered per playing position for each study. Figure 9 combines the studies where players were grouped as central defenders, external defenders, central midfielders, external midfielders, and forwards. Central midfielders (11,012 m), external midfielders (10,894 m) and external defenders (10,457 m) covered similar distances. Forwards (10,068 m) and central defenders (9598 m) covered substantially less distance than other positions.

**Fig. 10:**
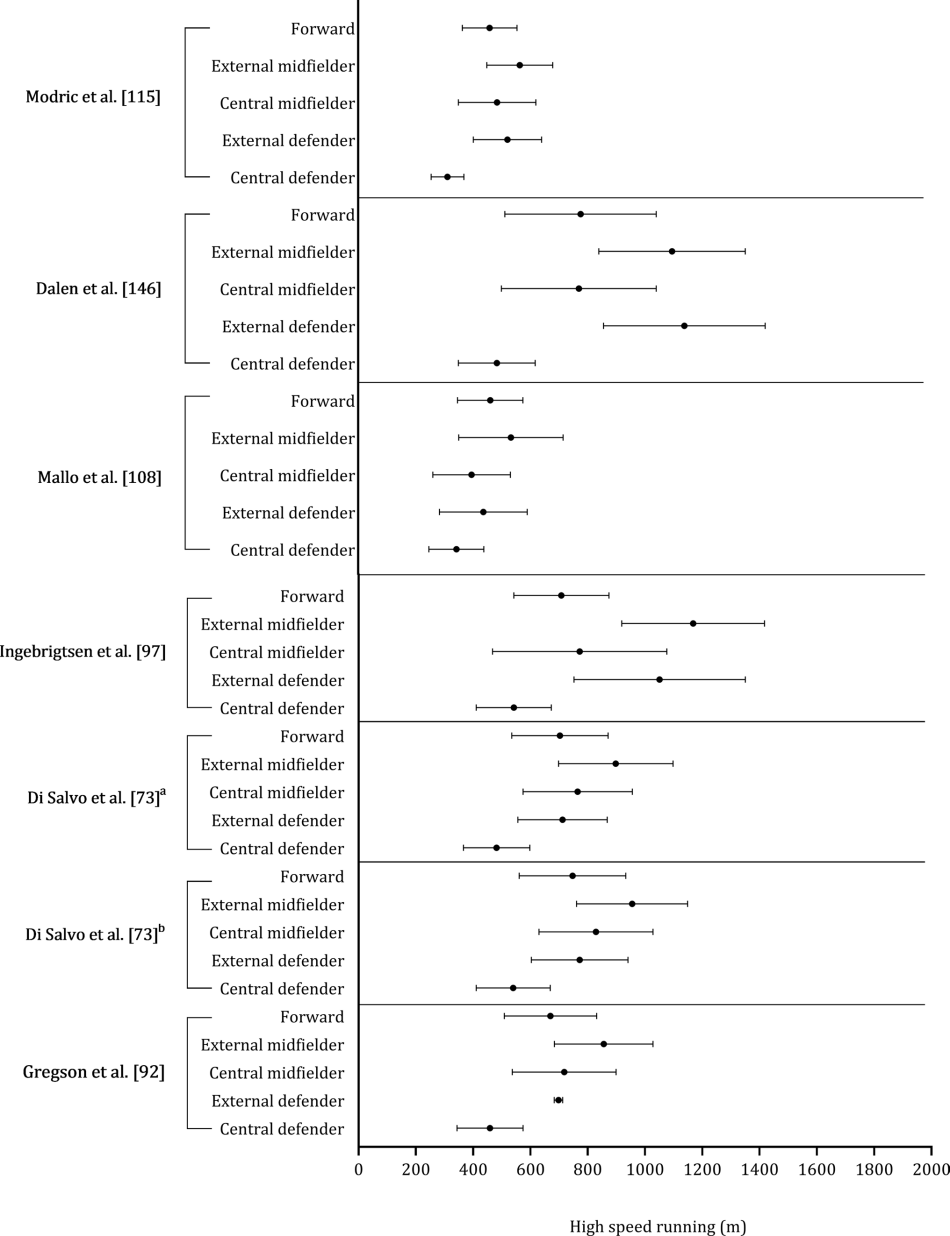
Descriptive statistics (mean ± standard deviation) of distance in high-speed running covered by playing position (central defender, external defender, central midfielder, external midfielder, forward). ^**a**^Championship League, ^b^Premiership

**Fig. 11:**
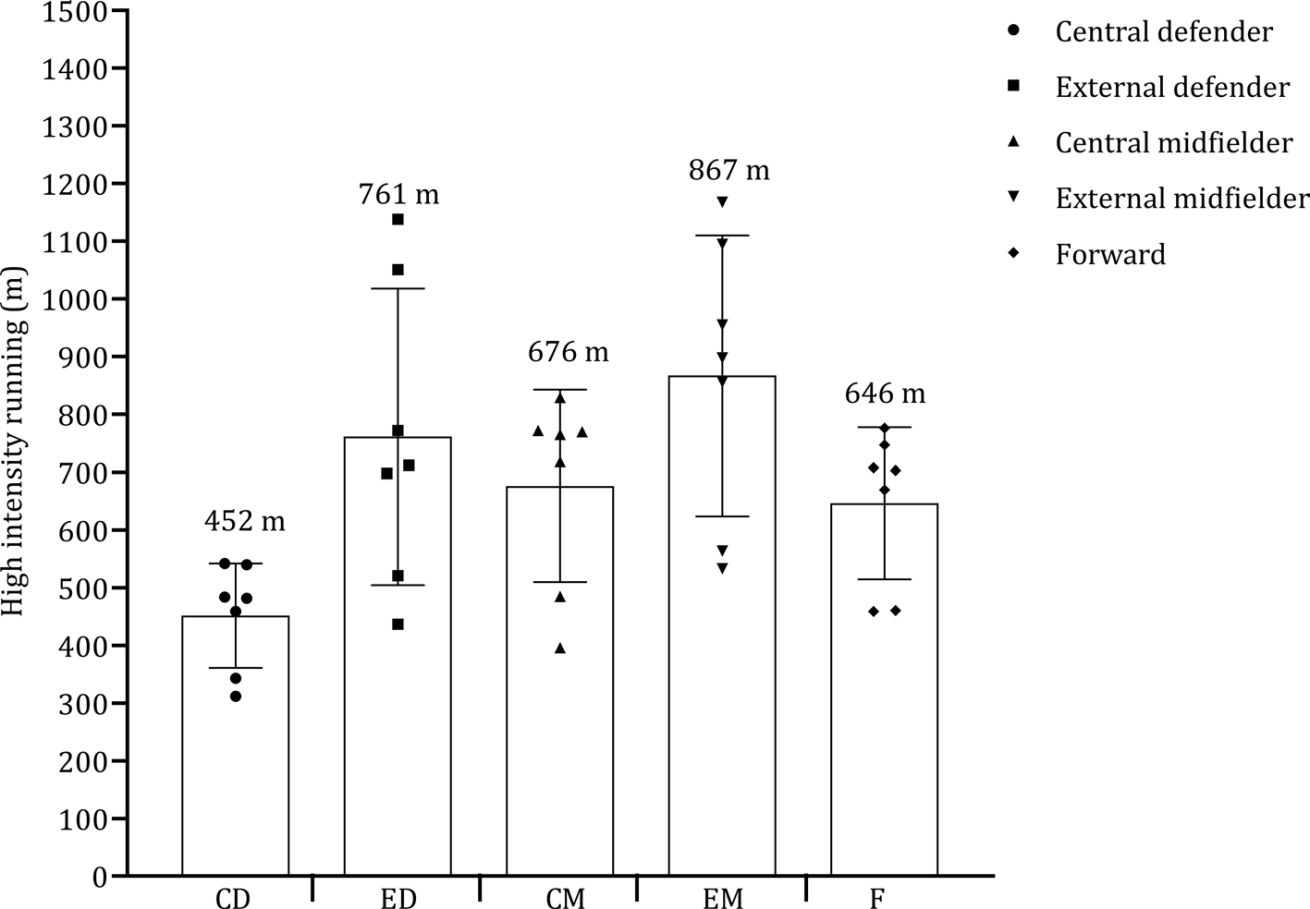
Pooled mean and standard deviation of studies that examined distance in high-speed running covered by playing position. Each symbol represents the mean of individual studies

**Fig. 12:**
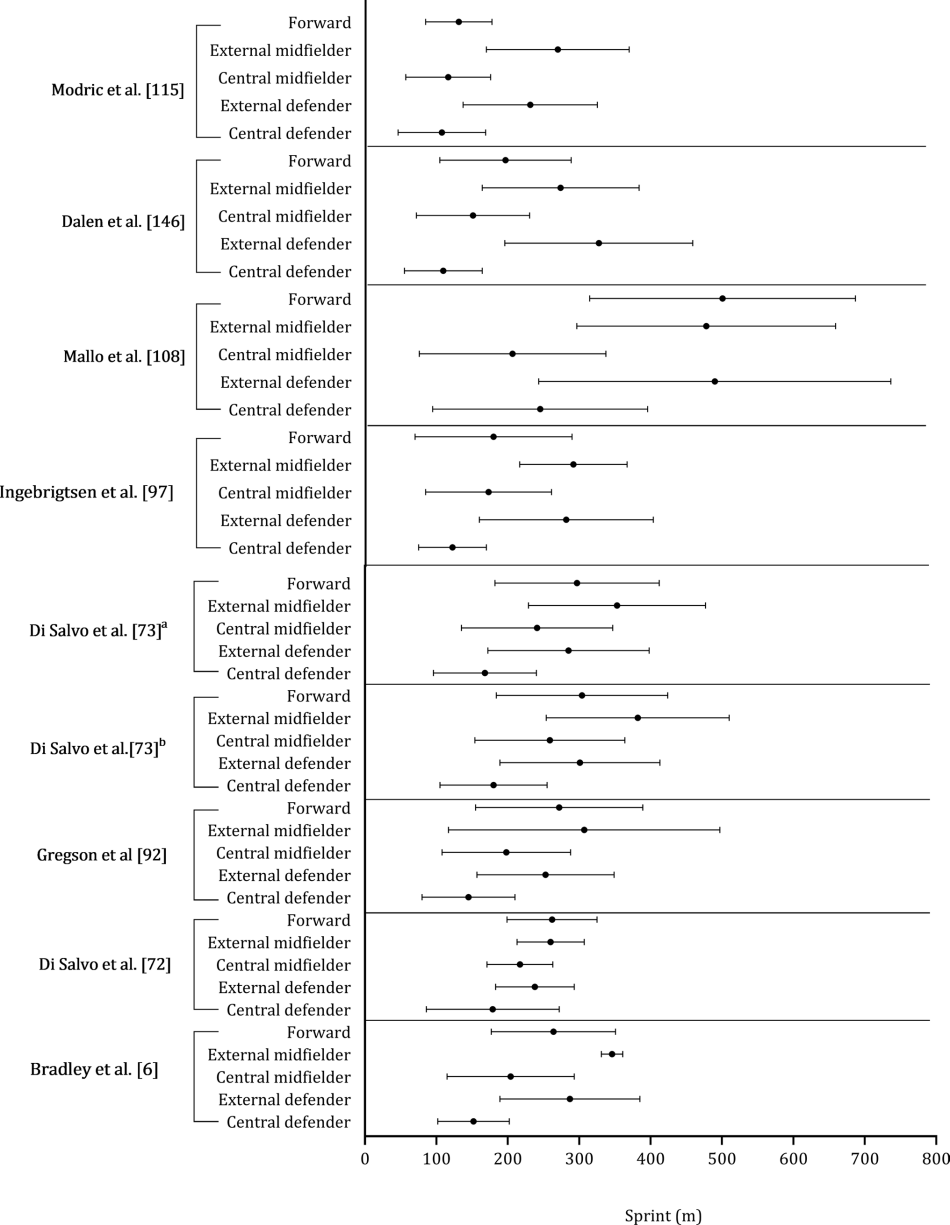
Descriptive statistics (mean ± standard deviation) of distance covered in sprint by playing position (central defender, external defender, central midfielder, external midfielder, forward).^**a**^Championship League, ^b^Premiership

**Fig. 13:**
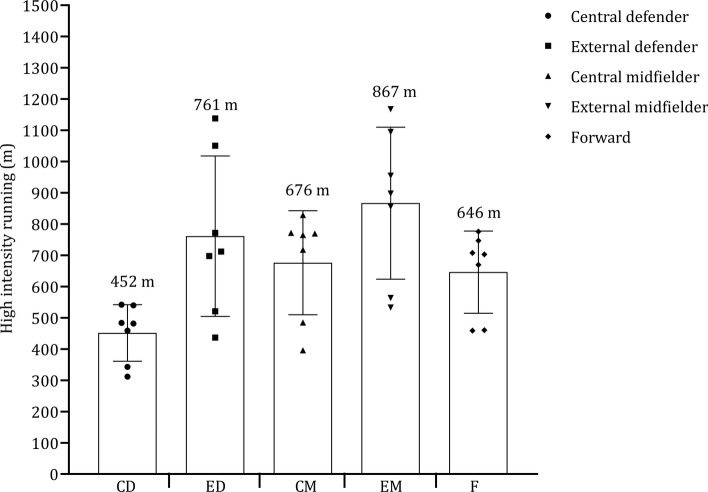
Pooled mean and standard deviation of studies that examined distance covered in sprint by playing position. Each symbol represents the mean of individual studies

Figure 10 shows the high-speed running distance covered by central defenders, external defenders, central midfielders, external midfielders, and forwards. Central defenders and forwards covered less distance at high-speed running than central midfielders, external defenders, and midfielders. In the seven studies included, external midfielders covered, on average, + 106 m and + 191 m more in high-speed running than external defenders and central midfielders, respectively (see Fig. 11). External defenders and midfielders cover more distance than central defenders, central midfielders, and forwards, as shown in Fig. 12. Sprint distance was higher in external midfielders (330 m) compared with external defenders (273 m), forwards (280 m), central midfielders (224 m), and central defenders (180 m), as illustrated in Fig. 13.

**Fig. 14:**
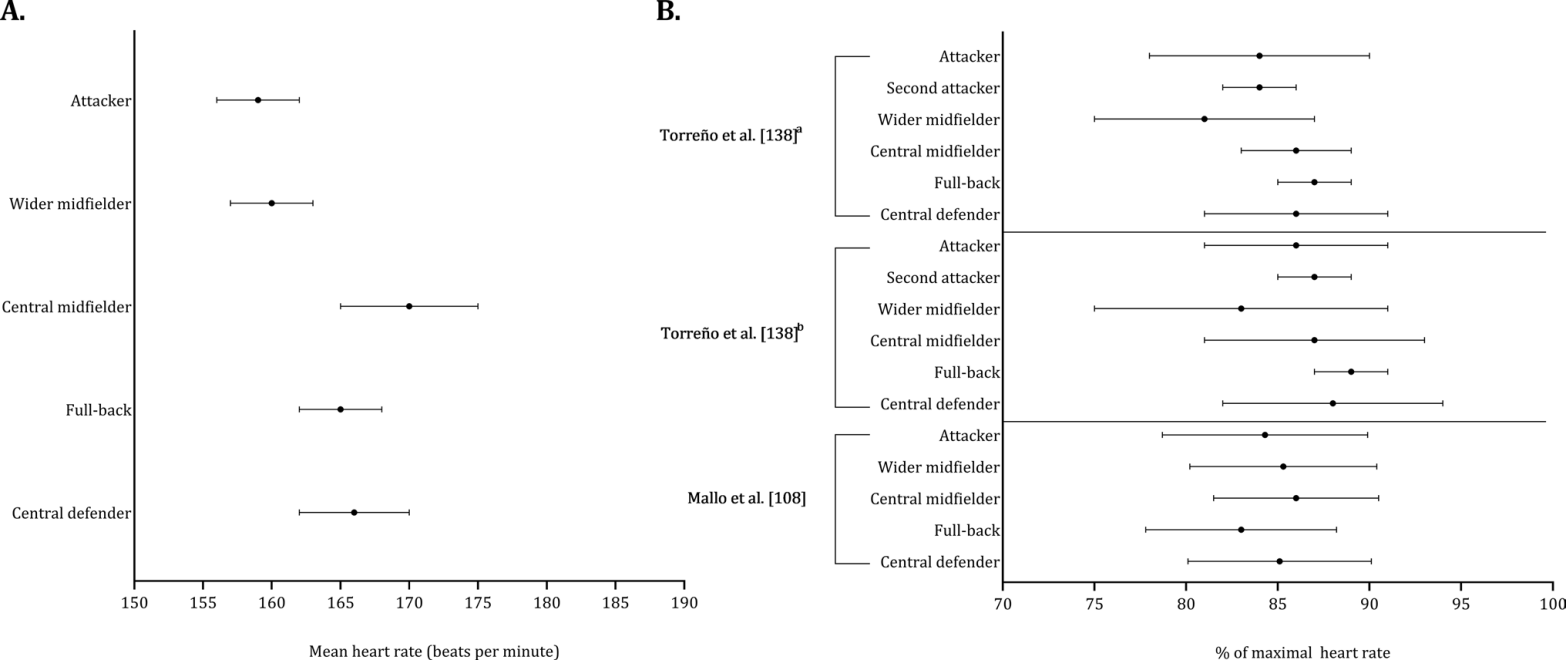
Descriptive statistics (mean ± standard deviation) of heart rate (absolute values: **A** (Bujnovsky et al. [52]) and relative heart rate (% of maximal heart rate: ** B**). ^a^First half, ^b^Second half

**Fig. 15:**
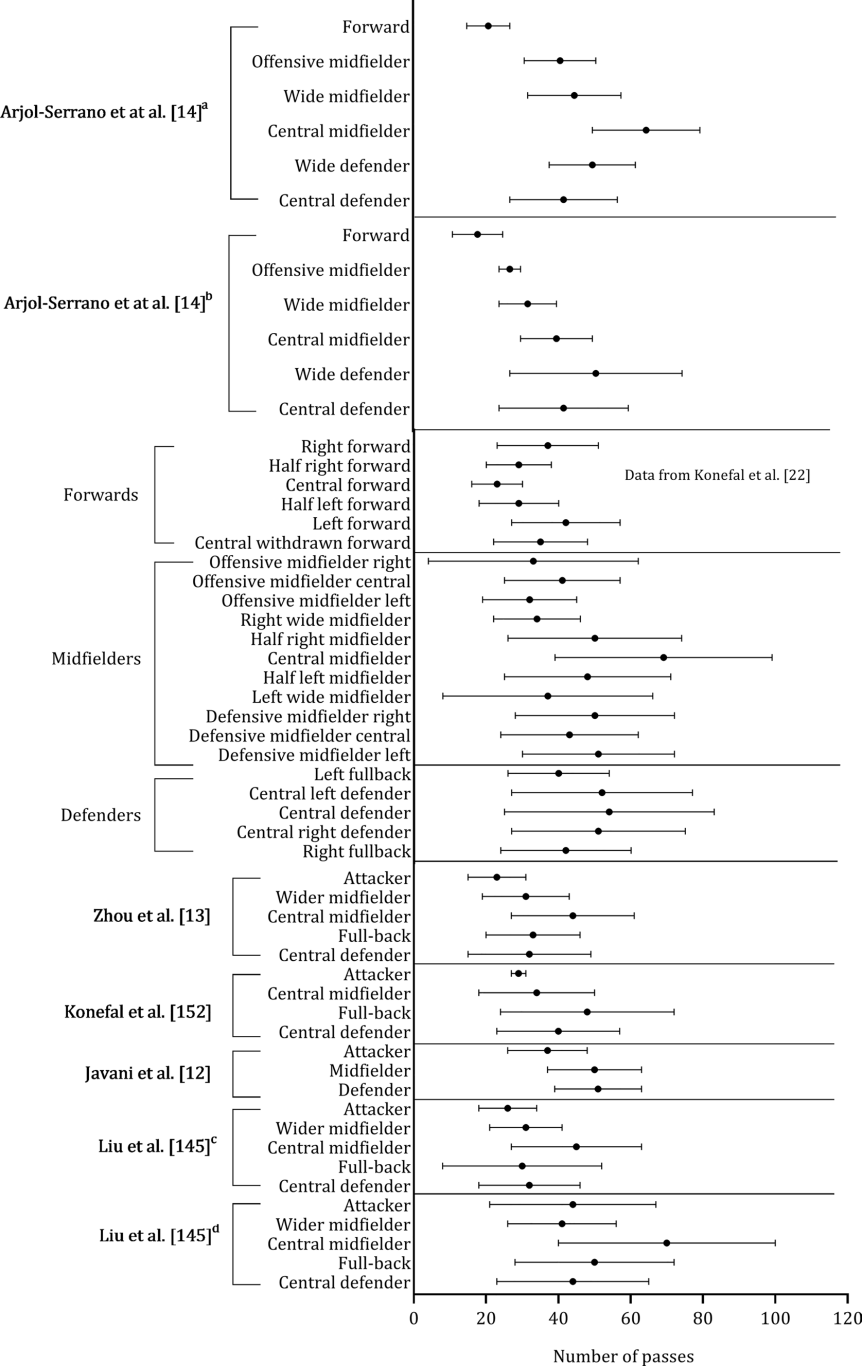
Descriptive statistics (mean ± standard deviation) of number of passes in each study. ^a^Considering the following tactical scheme: 1–4-4–2; ^b^Considering the following tactical scheme: 1–4-2–3-1; ^c^bottom three teams; ^d^top three teams

Mean heart rate, expressed in beats per minute or as a percentage of maximal heart rate, was obtained from three studies. These studies did not use the same classification to group players by playing position (Fig. 14, A and B). Although the playing position classification in studies that included the number of passes varied, defenders and midfielders appear to perform more passes than external midfielders and forwards (Fig. 15).

### Risk of Bias Assessment

**Fig. 16:**
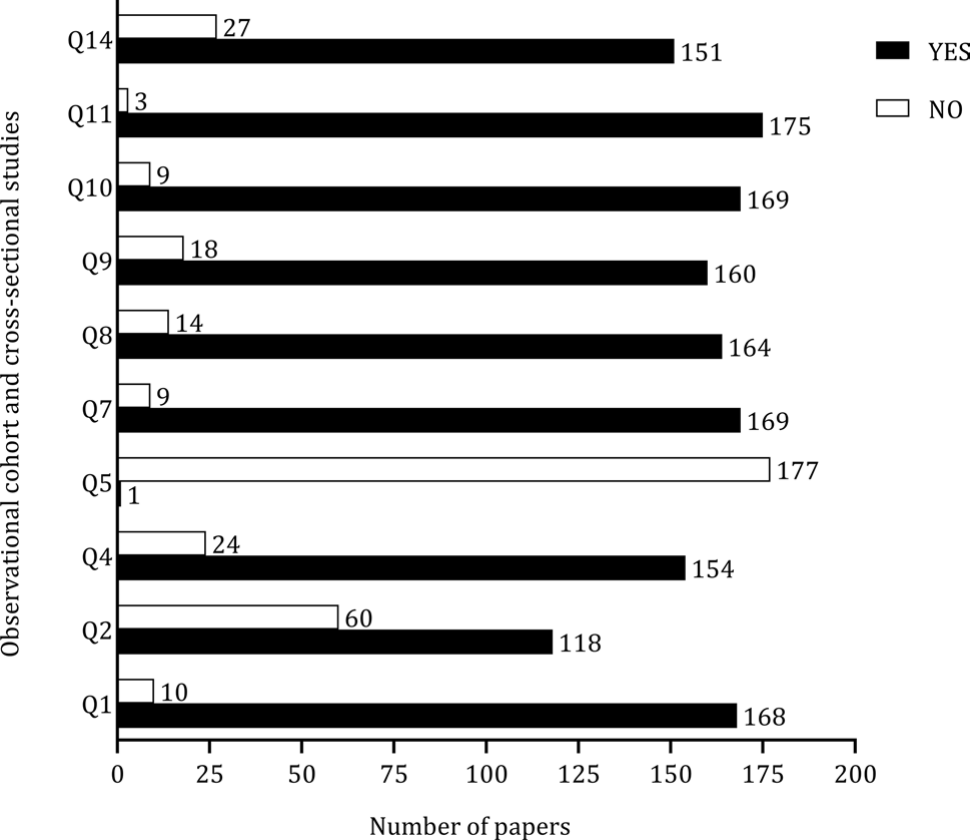
Summary of the quality of studies included in the present review. Q1: Was the research question or objective in this paper clearly stated? Q2: Was the study population clearly specified and defined? Q3: Was the participation rate of eligible persons at least 50%? Q4: Were all the subjects selected or recruited from the same or similar populations (including the same time period)? Were inclusion and exclusion criteria for being in the study prespecified and applied uniformly to all participants? Q5: Was a sample size justification, power description, or variance and effect estimates provided? Q6: For the analyses in this paper, were the exposure(s) of interest measured prior to the outcome(s) being measured? Q7: Was the timeframe sufficient so that one could reasonably expect to see an association between exposure and outcome if it existed? Q8: For exposures that can vary in amount or level, did the study examine different levels of the exposure as related to the outcome (e.g., categories of exposure, or exposure measured as continuous variable)? Q9: Were the exposure measures (independent variables) clearly defined, valid, reliable, and implemented consistently across all study participants? Q10: Was the exposure(s) assessed more than once over time? Q11: Were the outcome measures (dependent variables) clearly defined, valid, reliable, and implemented consistently across all study participants? Q12: Were the outcome assessors blinded to the exposure status of participants? Q13: Was loss to follow-up after baseline 20% or less? Q14: Were key potential confounding variables measured and adjusted statistically for their impact on the relationship between exposure(s) and outcome(s)?

The tool to examine the risk of bias and associated with Fig. 16 is presented in ESM 1. Questions 3, 6, 12, and 13 were not analyzed since they did not correspond with the studies of the present review. In 60 studies (33%), the sample was not clearly defined, and only one study clearly stated the power sampling (question 5). The description of independent variables was not evident in 19 studies (10%). The eligibility criteria comprised elite and professional soccer players; however, some studies combined samples from different competitive leagues and divisions, making it difficult to determine exact playing level. Twenty-seven studies (15%) did not consider these confounding factors.

## Discussion

The results of this systematic scoping review reveal a significant increase in playing positions over recent years, with more than half of the studies published after 2019. The majority of this research was conducted in Europe, mostly in Spain and England, with less representation from other continents. The findings suggest that central and external midfielders, and external defenders cover greater total and high-speed distance than forwards or central defenders. Another key finding was that sprint distance was higher in external midfielders versus all other positions. It was also reported that defenders and central midfielders appear to perform more passes than external midfielders and forwards. Although heart rate was the most reported physiological variable, there are few studies that characterize the physiological profiles of each positional role. The present findings provide a detailed description of the demands placed on elite soccer players, according to their positional role, which may be helpful in the development of individual training practices.

### Investigating Physical Performance Across Playing Positions

The observed disparities in distance covered by different playing positions, as depicted in Figs. 8 and 9, provide valuable insights into the distinct physical demands associated with each positional role. Central and external midfielders exhibited similar distances covered [38, 42, 44, 47, 50, 70, 73, 108, 144], with central midfielders averaging a distance of 11,012 m (range: 9202–12,261 m), and external midfielders averaging a distance of 10,894 m (range: 9538–12,320 m) when considered across all the relevant studies included in the current review. This similarity in distance can be attributed to their shared responsibilities in both offensive and defensive play, which requires that larger distances are covered [190].﻿ These key positions are also key in the transition, effectively bridging the gap between defense and attack [166].

The data reveals that external defenders covered an average distance of 10,457 m, which is slightly less than the distances covered by central and external midfielders. Modern-day fullbacks are no longer confined to defensive duties but are also actively involved in the attack, resulting in them covering a considerable distance during a match [191]. This adaptation can depend on the specific formation employed by the team and the tactical choices made by the coach during both defensive and offensive phases [190]. In contrast, forwards and central defenders covered substantially less distance [6, 42, 44, 47, 70, 73, 97, 146, 155], with forwards averaging 10,068 m (range: 8621–11,254 m) and central defenders covering 9598 m (range: 7525–10,627 m). This difference can be associated with the roles on the field, in that forwards primarily have the responsibility of leading attacks and creating goal-scoring opportunities, involving shorter but more explosive bursts of high-intensity accelerations [192]. Central defenders, on the other hand, are required to maintain a compact defensive structure, which involves less ground coverage and more positional play.

The data provided on high-speed running and sprinting distances across different playing positions sheds light on the varied physical demands of soccer roles [6, 73, 92, 115]. External midfielders complete more high-speed running than all other positions [73, 92, 97, 108, 115, 146], with a further 106 m and 191 m covered versus external defenders and central midfielders, respectively. External midfielders are often tasked with covering extensive ground at fast speeds during both defensive and offensive phases. Their ability to engage in high-speed running may also be crucial in their capacity to transition effectively and support both ends of the pitch [193]. Central defenders and forwards covered the least high-speed running distance compared with all other positions [73, 92, 97, 108, 146]. Central defenders typically adhere to positional roles focused on maintaining their position in the center of the field. Forwards aim to create space through frequent movements, often relying on shorter, more dynamic actions [91]. Midfielders and external defenders are tasked with establishing connections between different sectors of the field [194]. They do so by exploring various depths of the field and attempt to exploit spatial gaps in the opposition’s defensive line, which requires that they achieve higher speeds and cover longer distances [195]. This rationale could account for the greater distances at higher speed thresholds [193].

External midfielders perform the greatest sprint distance (330 m), followed by external defenders (273 m), forwards (280 m), central midfielders (224 m), and central defenders (180 m). This might be attributed to the notion that external players are often isolated in wide positions in one-on-one scenarios, which requires maximal sprints to ‘out compete’ the opponent. Another explanation is that wide midfields are responsible for both defensive and offensive duties [73, 92, 97, 108, 115, 146] and sprints might be crucial to the success of this position as straight line sprints often precede goals [196].

The data presented in this scoping review highlight the distinctive physical demands associated with different soccer playing positions. Central and external midfielders, along with external defenders, covered substantial distances during matches. Forwards and central defenders covered less ground, given their tactical discipline and more specialized responsibilities. However, it is essential to consider that performance can vary depending on factors such as tactical formation, competitive level, playing style, and the team's adopted model. These insights offer valuable information for tailoring position-specific training and enhancing player performance. Future research should further enrich these findings by incorporating combined tactical information to elucidate how behavior influences team performance [197], addressing a gap in the literature that warrants attention.

### Methodological Trends in Exploring Differences Between Playing Positions

In this scoping review of 178 studies, the classification of players according to their playing positions reveals some interesting trends and discrepancies. Over half of the studies in the current review (*n* = 105, ~ 59%) employed a categorization system that encompassed central defenders, full-backs, central midfielders, wide midfielders, and forwards. This approach represents a consistent methodological approach to categorizing player roles, allowing for an accurate examination of various playing positions [99]. However, approximately 16% of the studies (*n* = 29) omitted the inclusion of full-backs and external midfielders in their classification systems. This omission has potential ramifications for the analytical sensitivity of the research, primarily because it is consistently observed that central defenders cover significantly shorter distances than fullbacks [36, 42, 70]. Discrepancies are also observed between the playing styles of external midfielders and central midfield players. The classification methodologies employed by a significant subset of studies (*n* = 27, ~ 15%) demonstrated substantial variability in playing position [55, 61, 120, 148], highlighting the lack of consensus or standardization in categorizing playing positions within the soccer research community. These findings explain the need for improved consistency in the classification of playing positions in soccer research.

Goalkeepers are consistently excluded from research analyses, a trend that may be attributed to the unique nature of their position. Due to the nature of their role, goalkeepers experience fewer physical, physiological, and technical demands. This exclusion from research raises questions about the adequacy of our understanding of goalkeepers' contributions and challenges within the broader context of the game. Investigations into the profiles of this unique position would enhance the understanding and training practices of goalkeepers.

The technologies employed for the measurement of activity during matches have evolved over recent years. The use of automatic or semi-automatic video-based systems was particularly prevalent [21, 82, 99, 130], with 78 papers (52%) adopting these technologies. Microelectromechanical systems (MEMS) were utilized in 66 papers [40, 43, 44], highlighting the increasing integration of modern technologies, attributed to a change in FIFA rulesets. The focus on specific metrics was evident in the studies reviewed, with significant attention directed towards parameters such as total distance covered (109 papers; 45%), high-speed running (62 papers; 25%), and sprinting (73 papers; 30%). The prevalence of video-based systems and notational analysis has increased in the investigation of technical and tactical aspects of soccer [22, 128, 161]. However, it is important to note that despite these advancements in technology, there is a noticeable lack of integrated research that combines physical, physiological, technical, and tactical analyses, particularly across playing positions [197]. Addressing this gap by working collaboratively and in an inter-disciplinary manner would enhance our understanding of how positional profiles influence match outcomes.

Two studies assessed the physiological differences in playing positions [52, 85]. The discrepancy in quantity of studies assessing physical performance and physiological characteristics might be attributed to the difficulties associated with collecting physiological data during matches. This might have potential implications for practice given that internal physiological load does not appear to correlate with external load [198]. The nuanced relationship between external and internal loads involves complex and multifaceted factors influencing player performance and wellbeing that extend beyond locomotion activities. Assessing physiological load can offer valuable insights into player management and training strategies, ultimately advancing our understanding of the physiological profiles of different playing positions.

### Evolving Trends in Analyzing Soccer Playing Positions

The present review article highlights a marked increase in research output specific to player positions over the past 5 years (2019–2023), with 54% of the studies being published during this time frame. This trend might be attributed to the well-established practice of monitoring the demands of match play in applied soccer clubs [199, 200]. It appears that there is an increasing trend of practitioners and researchers collaborating in an attempt to better use applied data to enhance understanding of various elements of soccer, including the demands of each positional role.

The location of soccer research on the topic of playing positions has mainly originated from Europe, specifically from Spain (*n* = 41; 27%) and England (*n* = 25; 15%). One potential explanation could be the strong tradition of soccer in Europe, where the sport is deeply ingrained in the culture [201]. This long-standing soccer heritage might naturally lead to a greater interest and investment in research in these countries. The presence of top-tier soccer leagues (e.g., United Kingdom, Spain, Italy, Germany, France) [202] in these nations could provide further opportunities for research collaboration and funding, which may in turn perpetuate the wealth of research in Europe. However, although Spain and England possess some of the highest-level soccer teams and sport science institutions in Europe, the current data and understanding surrounding playing positions in soccer might be solely reflective of the practices and cultures within these specific countries. Determining the positional differences from an array of continents might enhance understanding of playing positions in soccer on a greater scale.

### Methodological Limitations and Future Directions

There are several methodological limitations present within the literature. There is a lack of clear sample definitions, limited reporting of power analyses, insufficient descriptions of independent variables and ambiguity surrounding the playing levels of participants across studies. This leads to confusion, a lack of consensus of surrounding data interpretation, and makes it difficult to draw conclusions that are representative, as players in certain positions at the elite level are likely to demonstrate divergent activity profiles versus a lower-level soccer player. The failure to address contextual factors in a substantial portion of the reviewed studies highlights the need for a greater understanding of applied practices in soccer. For instance, it remains unclear as to what extent positional roles are influenced by substitution strategies, as none of the included studies separated the analyses to account for partial match players. It is likely that if players enter or leave the field part way through a match, these profiles are likely to differ from those of players that play an entire soccer match [203]. It might also need to be considered how the requirements of each playing position are influenced by fixture congested scenarios [204]. Fixture congested periods are a major contemporary challenge faced by soccer teams and must be considered in future work characterizing position differences to ensure coaches are better informed as to which positions should be managed due to higher propensities of underperformance and injury [205].

## Conclusion

The scoping review aimed to compare the impacts of playing position on physical, physiological, and technical demands in soccer matches. The findings demonstrate that central and external midfielders, and external defenders, cover greater total and high-speed distance than forwards or central defenders. Sprint distance was higher in external midfielders versus all other positions. Defenders and central midfielders perform more passes than external midfielders and forwards. These data might support coaches and practitioners in preparing players for competition demands through the development of individualized training practices that allow athletes to better handle specific positional demands.

## Supplementary Information

Below is the link to the electronic supplementary material.

Supplementary file1 (DOCX 16 KB)

Supplementary file2 (DOCX 21 KB)

## Declarations

### Availability Data and Material

The authors confirm that the data supporting the findings of this study are available within the article and its supplementary materials.
