# Supplementary material for: The Influence of Playing Position on Physical, Physiological, and Technical Demands in Adult Male Soccer Matches: A Systematic Scoping Review with Evidence Gap Map
Source: Sports Med. 2024 Sep 11;54(11):2841–64. doi: 10.1007/s40279-024-02088-z (PMC11561100; doi:10.1007/s40279-024-02088-z)
Supplement: Supplementary file 2 — Supplementary file2 (DOCX 21 KB) [file 40279_2024_2088_MOESM2_ESM.docx]

| **Supplementary Material 2.** Compilation of studies that used similar thresholds^†#^. | | |
| --- | --- | --- |
| Thresholds | References |  |
| Standing (0 km^.^h^-1^), walking (6 km^.^h^-1^), jogging (8 km^.^h^-1^), low speed running (12 km^.^h^-1^), moderate speed running (15 km^.^h^-1^), high speed running (18 km^.^h^-1^), sprinting (30 km^.^h^-1^) and backward running (10 km^.^h^-1^) | (Mohr et al., 2003; Barros et al., 2007) |  |
| Standing, walking, jogging (0-11 km^.^h^-1^), low speed running (11.1–14 km^.^h^-1^), moderate-speed running (14.1–19 km^.^h^-1^), high speed running (19.1–23 km^.^h^-1^), sprinting (> 23 km^.^h^-1^), accelerations (> 1 m^.^s^-1^), deaccelerations (<1 m^.^s^-1^) | (Di Salvo et al., 2007, Carling et al., 2010; Lagos-Penas et al., 2011; Morera-Barrantes et al., 2021) |  |
| Standing (0–0.6 km^.^h^-1^), walking (0.7–7.1 km^.^h^-1^), jogging (7.2–14.3 km^.^h^-1^), running (14.4–19.7 km^.^h^-1^), high speed running (19.8–25.1 km^.^h^-1^), sprinting (>25.1 km^.^h^-1^), high intensity running (running speed >14.4 km^.^h^-1^), very high-intensity running (running speed >19.8 km^.^h^-1^) | (Bradley et al., 2009; Di Salvo et al., 2009; Bradley et al., 2010; Gregson et al., 2010; Di Salvo et al., 2010; Bradley et al., 2011; Bradley et al., 2013a; Bradley et al., 2013b; Rampini et al., 2007; Di Mascio et al., 2013; Di Salvo et al., 2013; Gaudino et al., 2013; Ingebrigtsen et al., 2015; Mallo et al., 2015; Dalen et al., 2016; Varley et al., 2018; Baptista et al., 2018; Martin Fuentes et al., 2021; Teixeira et al., 2021; Radziminski et al., 2022) |  |
| Light speed (0.0–11.0 km^.^h^-1^), low speed (11.1–14.0 km^.^h^-1^), moderate speed (14.1–19.0 km^.^h^-1^), high-speed and sprinting (>19.1 km^.^h^-1^) | (Carling, 2010) |  |
| Standing, walking (0-11 km^.^h^-1^), jogging (11.1-14 km^.^h^-1^), running (14.1-17 km^.^h^-1^), fast running (17.1-21 km^.^h^-1^), high-speed running (21-23.9 km^.^h^1^), sprinting (>24 km^.^h^-1^) | (Dellal et al., 2010; Dellal et al., 2011; Andrzejewski et al., 2012; Andrzejewski et al., 2013; Andrzejewski et al., 2014; Andrzejewski et al., 2016b; Miñan-Espino et al., 2017; Andrzejewski et al., 2019; Chmura et al., 2018; de Silva et al., 2017;) |  |
| Walking (< 5 km^.^h^-1^), jogging (5 – 13 km^.^h^-1^), speed below the anaerobic threshold (13 – 16 km^.^h^-1^), speed above the anaerobic threshold (16 – 19 km/h), and sprint (> 19 km^.^h^-1^) | (Vigne et al., 2010; Eirale et al., 2011; Digne et al., 2013) |  |
| Low-to-moderate intensity (0.0-14.3 km^.^h^-1^); high intensity (14.4-19.7 km^.^h^-1^), and very high intensity (>19.8 km^.^h^-1^) | (Carling, 2011; Carling et al., 2012) |  |
| Low intensity activity (speed movment < 10 km^.^h^-1^), medium intensity activity (time between 10 and 15 km^.^h^-1^), high intensity activity (> 15 km^.^h^-1^) | (Josak et al., 2011; Ademovic, 2016; Aquino et al., 2017; Vieira et al., 2018) |  |
| Standing/walking (0-6.9 km^.^h^-1^), jogging (7.0-12.9 km^.^h^-1^), running (13.0- 17.9 km^.^h^-1^), high-speed running (18.0-20.9 km^.^h^-1^), sprinting (>21 km^.^h^-1^); accelerations: 1.0-1.5 m^.^s^-2^, 1.6-2.0 m^.^s^-2^, > 2.0-2.5 m^.^s^-2^, >2.5 m^.^s^-2^) | (Casamichana et al., 2013) |  |
| Stationary–walking (0–3.9 km^.^h^-1^), jogging (4.0–6.9 km^.^h^-1^), quick running (7.0–12.9 km^.^h^-1^), high-intensity running (13.0–17.9 km^.^h^-1^) and sprint (>18 km^.^h^-1^) | (Domene, 2013) |  |
| Light (18-21 km^.^h^-1^), high (21.1-23.0 km^.^h^-1^), very high (23.1-25 km^.^h^-1^), sub-maximal (25.1-27 km^.^h^-1^), and maximal intensity running (27.1 km^.^h^-1^) | (Djaoui et al., 2014) |  |
| walking (0.1-7.0 km^.^h^-1^), running at low-speed (7.1-13.0 km^.^h^-1^), at medium-speed (13.1-18.0 km^.^h^-1^), at high-speed (18.1-21.0 km^.^h^-1^), and at sprint (>21.0 km^.^h^-1^). | (Suarez Arrone et al., 2015; Al Haddad et al., 2017) |  |
| low-intensity (≤ 14.4 km^.^h^-1^), high-intensity (14.4>km^.^h^-1^) | (Andrzejewski et al., 2016a) |  |
| Light intensity running (0.0–14.0 km^.^h^-1^); moderate intensity running (14.1–19.0 km^.^h^-1^); high-intensity running (19.1-23.0 km^.^h^-1^); sprint (23.1 km^.^h^-1^) | (Soroka et al., 2016;) |  |
| above 13 km^.^h^-1^; above 18 km^.^h^-1^ | (Torreño et al., 2016;) |  |
| High speed (> 15 km^.^h^-1^), high accelerations (3 m^.^s^-2^) | (Hoppe et al., 2017) |  |
| sprint (> 22.68 km^.^h^-1^) | (Andrzejewski et al., 2017) |  |
| low intensity (0-14.0 km^.^h^-1^), medium intensity (14-21 km^.^h^-1^), high intensity running (> 21 km^.^h^-1^), very high intensity running (21-24 km^.^h^-1^), sprint (> 24 km^.^h^-1^) | (Padrón-Cabo et al., 2018; Lago-Penas et al., 2021; Lorenzo-Martínez et al., 2021a) |  |
| low intensity (0-14.0 km^.^h^-1^), average intensity (14.1-19.8 km^.^h^-1^), high speed running (19.9-25.2 km^.^h^-1^), sprint (>25.2 km^.^h^-1^) | (Soroka, 2018; Modric et al., 2021; Vilamitijana et al., 2021; Modric et al., 2022a; Modric et al., 2023a) |  |
| High speed running (> 19.8 km^.^h^-1^) | (Dalen et al., 2019; Ju et al., 2023; Modric et al., 2023b; Hills et al., 2020, Modric et al., 2022b) |  |
| very high speed running (100% maximal aerobic speed-30% anaerobic speed reserve), sprint (> 30% anaerobic speed reserve), moderate intensity acceleration (50-75% maximal accelerative capacity), high-intensity acceleration (> 75% of maximal accelerative capacity) | (Abbot et al., 2018) |  |
| high-speed running(> 16 km^.^h^-1^), high acceleration (> 2 m^.^s^-2^), high deceleration (< -2 m^.^s^-2^ | (Altavilla et al., 2017; Altavilla et al., 2018; Filleti et al., 2019) |  |
| Above 20 km^.^h^-1^, below 20 km^.^h^-1^ | (Conde et al., 2018) |  |
| High speed running (> 19.8 km^.^h^-1^), sprint (> 25.2 km^.^h^-1^), high intensity accelerations (> 3 m^.^s^-2^), high intensity decelerations (< -3 m^.^s^-2^) | (Martín-Garcia et al., 2018; Mitrotasios et al., 2021) |  |
| High speed running (> 21.0 km^.^h^-1^) | (Baptista et al., 2018; Ponce-Bordon et al., 2021) |  |
| Three different speed zones: 12 km^.^h^-1^, 18 km^.^h^-1^, 24 km^.^h^-1^ | (Gonçalves et al., 2021, Lobo-Araujo et al., 2021) |  |
| Speed (> 18.1 km^.^h^-1^), sprint (22.7 km^.^h^-1^) | (Lorenzo-Martínez et al., 2021b) |  |
| Walking (0-5.99 km^.^h^-1^), jogging (6-11.9 km^.^h^-1^), running (12.0-15.7 km^.^h^-1^), high intensity running (15.8-19.6 km^.^h^-1^), fast running (19.7-23.7 km^.^h^-1^), sprint (> 23.8 km^.^h^-1^) | (Metaxas, 2021) |  |
| 0-6.99 km^.^h^-1^, 7.00-14.99 km^.^h^-1^, 15.00-19.99 km^.^h^-1^, 20.00-25.99 km^.^h^-1^, accelerations: < 3^.^m^-2^, 2.00-2.99 m^-2^, 1.00-1.99 m^-2^, > 3^.^m^-2^ | (Praça et al., 2021) |  |
| low intensity running (< 14.3 km^.^h^-1^), running (14.4-19.7 km^.^h^-1^), high intensity running (> 19.8 km^.^h^-1^), accelerations (> 0.5 m^.^s^-2^), high intensity accelerations (> 3 m^.^s^-2^), decelerations ( < - 0.5 m^.^s^-2^), high intensity decelerations (< - 3 m^.^s^-2^) | (Sekulic et al., 2021) |  |
| Walking (0-7.2 km^.^h^-1^), jogging (7.2-14.3 km^.^h^-1^), running (14.4-20.0 km^.^h^-1^), high speed running (> 20 km^.^h^-1^) | (Yuksel et al., 2022) |  |
| >14 km^.^h^-1^, >21 km^.^h^-1^, >24 km^.^h^-1^ | (Asian-Clemente et al., 2022) |  |
| Sprint (≥ 25.2 km^.^h^-1^) | (Caldbeck et al., 2022) |  |
| Low speed running (0-10.8 km^.^h^-1^), intermediate speed running (10.8-19.8 km^.^h^-1^), high speed running (19.9-25.2 km^.^h^-1^), sprint (> 25.2 km^.^h^-1^) | (Djaoui et al., 2021) |  |
| < 10.8, 10.8-19.7, 19.8-25.2, 25.2, acceleration (≥ 2 m^.^s^-2^), deceleration (≤ -2 m^.^s^-2^) | (Dos Santos et al., 2022) |  |
| High intensity (17-23.9 km^.^h^-1^), sprint (> 24.0 km^.^h^-1^) | (Forcher et al., 2022) |  |
| High intensity (14-21 km^.^h^-1^), very high intensity (> 21.0-24 km^.^h^-1^), sprint (> 24 km^.^h^-1^) | (Garcia Aliaga et al., 2022) |  |
| Low speed running(< 14 km^.^h^-1^), medium speed running (14-18 km^.^h^-1^), high speed running (18-21 km^.^h^-1^), very high speed running (21-24 km^.^h^-1^), sprint (> 24 km^.^h^-1^), acceleration (≥ 2 m^.^s^-2^), deceleration (≤ -2 m^.^s^-2^) | (Guerrero-Calderon et al., 2022) |  |
| 20-25 km^.^h^-1^, > 25 km^.^h^-1^ | (Ortega et al., 2022) |  |
| High speed running (15-20 km^.^h^-1^), very high speed running (20-24 km^.^h^-1^), sprint (24 km^.^h^-1^), accelerations (> 3 m^.^s^-2^), decelerations (< -3 m^.^s^-2^) | (Riboli et al., 2021) |  |
| Low speed running (6-12 km^.^h^-1^), medium speed running (12-18 km^.^h^-1^), high speed running (18-21 km^.^h^-1^), very high speed running (21-24 km^.^h^-1^), sprint (>24.1 km^.^h^-1^) | (Castillo-Rodriguez et al., 2023) |  |
| High speed running (19.8-25.2 km^.^h^-1^) | (Long et al., 2023) |  |
| † Organization of thresholds did not consider variations of 0.1 in different entry speeds.  # Some studies reported the values in m^.^s^-1^ and were converted to km^.^h^-1^. | | |
